# How Did COVID-19 Affect Mental Health and Access to Care in Persons With Inflammatory Bowel Disease

**DOI:** 10.1093/jcag/gwad033

**Published:** 2023-10-10

**Authors:** Seth R Shaffer

**Affiliations:** Section of Gastroenterology, Department of Internal Medicine, Max Rady College of Medicine, Rady Faculty of Health Sciences, University of Manitoba, Winnipeg, MB, Canada; University of Manitoba IBD Clinical and Research Centre, University of Manitoba, Winnipeg, MB, Canada

## Abstract

The coronavirus disease pandemic globally affected public health and the world economy, leading to an increase in mental health symptoms, thought to be due in part to periods of quarantining, restrictions, and other interventions used to curb ongoing transmission of the virus. It is well established that persons with inflammatory bowel disease (IBD) have significantly higher rates of depression and anxiety than the general population and that mental health symptoms can exacerbate disease severity. For persons with IBD, psychological distress was correlated with challenges in accessing medical care. In the early stages of the pandemic, endoscopy suites were closed, leading to fewer colonoscopies, although this rebounded the following year. This likely led to fewer diagnoses of IBD initially as people avoided the health care system, and also a reduction in IBD-related dysplasia being detected during colonoscopy. Many hospitals and health care clinics adjusted by delivering telemedicine for ambulatory care. Persons with IBD had increased stress about accessing both their health care provider and gastroenterologist during the pandemic, although many had increased satisfaction with the level of care they received virtually. Telemedicine is now being used in most clinics in conjunction with in-person care, to help deliver care, and can be cost-effective. Additional research is needed to assess whether heightened levels of mental health symptoms have led to worsening disease activity, and further, if a delay in health care access including colonoscopies and surgeries, or the perceived decreased access to health care professionals for some will have detrimentally affected the disease course for persons with IBD.

## Introduction

Coronavirus disease (COVID-19) is a viral infection that began in Wuhan, China, initially infecting people at the end of 2019 and shortly after spreading worldwide. On March 11, 2020, the World Health Organization declared the COVID-19 a global pandemic.^1^ The pandemic globally affected public health and the world economy, leading to an increase in mental health symptoms, thought to be due in part to periods of quarantining, restrictions, and other interventions used to curb ongoing transmission of the virus.^2^ Much of the mental health sequelae included anxiety and depression.^3,4^ For individuals with chronic diseases or other health concerns, psychological distress has related to challenges in accessing medical care including procedures, hospitalizations, and/or surgeries that would have otherwise been warranted in their disease management.^5^

Psychological distress, depression, and anxiety were found to be significantly elevated during the first wave of the COVID-19 pandemic compared with baseline rates^6^ and remained elevated in subsequent waves.^7,8^ A survey of Canadians revealed that over one-third had worse mental health during the first wave of the pandemic compared with before the pandemic, and roughly 40 percent of those who quarantined also reported worsening mental health symptoms.^2^

Inflammatory bowel disease (IBD) (including Crohn’s disease [CD] and ulcerative colitis [UC]) is a chronic immune-mediated inflammatory disease often requiring immunomodulatory and/or corticosteroid medications, hospitalizations for medical or surgical management, with high rates of Emergency Department use.^9^ It is well established that persons with IBD have significantly higher rates of depression and anxiety than the general population and that mental health symptoms exacerbate disease severity.^10–14^ Given the strong relationship between mental and physical health in IBD, increased mental health challenges are anticipated to translate to worsening disease outcomes.

Health care utilization was affected early on in the pandemic, as health care delivery was forced to adapt to prevent the transmission of the severe acute respiratory syndrome coronavirus 2 (SARS-CoV-2) virus. This lead to the use of telemedicine for some outpatient clinics, and personal protective equipment for in-person appointments and throughout hospitals. IBD-related procedures were affected as well, as most places saw these decrease in the early stages of the pandemic. Many persons with IBD had increased stress regarding accessing their health care providers, although a majority of people were satisfied with the care they received through telemedicine clinics. Access to care was defined by IBD-related outpatient visits, hospitalizations, endoscopies, and surgeries, and the definition of health care use can be challenging as it can often vary from article to article. Data on IBD-medication use or changes, along with primary care visits, were not included in this article.

The aim of this paper was to review how the mental health of persons with IBD was altered, along with how their access to care was affected, during the COVID-19 pandemic.

## Mental health symptoms

During the COVID-19 pandemic, much of the population, including those with IBD, had elevated mental health symptoms. This was especially prominent early on in the pandemic, as high levels of distress were seen around the world, among the general population.^15–17^ A Canadian survey revealed that the rate of anxiety increased fourfold compared with pre-pandemic levels (20 percent vs. 4 percent), while depressive symptoms more than doubled (10 percent vs. 4 percent).^18^ It was thought that perhaps this increase in mental health symptoms was due to uncertainty surrounding the virus, fear, financial insecurity, and restrictions leading to lockdowns and social isolation.^19^ After an initial increase in mental health symptoms early on in the pandemic, it was felt that some of these symptoms settled down, perhaps, due to changes in one’s perception of risk toward the COVID-19 virus.^20^

Persons with IBD are already at an increased risk of mental health disorders^10–14^ due to the chronicity of their illness, the unpredictability of onset of symptoms, and worries regarding disease and treatment outcomes or complications.^21^ There is also an increased risk of mental health disorders for years prior to the onset of disease, suggesting that mental health diseases may share biological underpinnings with IBD. Given the strong relationship between mental and physical health in IBD, increased mental health challenges are expected to translate to worsening disease outcomes, as active mental health symptoms can lead to worsened IBD outcomes^22^ and higher hospitalization rates.^23^

Relating to the COVID-19 pandemic, we have seen an increase in clinically significant mental health symptoms. A study from China surveyed over 2,200 persons with IBD showed that more than 50 percent of persons reported some degree of mood changes and 58 percent were worried about the risk of acquiring COVID-19 for themselves or their family.^24^ Harris et al.^25^ performed a survey of IBD patients in the UK, and a majority of the 685 respondents had significantly increased perceived stress, and many reported that COVID-19 had a negative impact on their quality-of-life. A survey of 693 persons with IBD in Spain reported rates of depression, anxiety, and stress as 11 percent, 20 percent, and 18 percent, respectively, during a COVID-mandated lockdown.^26^ Special populations such as persons who were pregnant or planning on becoming pregnant were affected as well. A survey of 73 pregnant women with IBD revealed that over half of them reported an increase in stress and depression symptoms during the pandemic, and half of those planning pregnancy reported that the pandemic significantly affected their pregnancy planning.^27^

Another survey study revealed that 40.5 percent of respondents reported worsening sleep, with 63.7 percent reporting worse stress.^28^ A survey study from Japan assessing 3,032 persons reported that the top causes of anxiety were contracting COVID-19 during hospital visits and contracting the virus because of their IBD or because of their IBD therapy.^29^

We performed a large survey study of persons with IBD within Manitoba, with a response rate of 47.1 percent (1,384 respondents).^30^ The validated measures of mental health symptoms were used, including the Perceived Stress Scale-10^31^ to measure perceived stress, Short Health Anxiety Inventory-14^32^ to measure health anxiety, Patient Health Questionnaire-9^33^ to measure depression, and Generalized Anxiety Disorder-7 (GAD-7)^34,35^ to measure generalized anxiety. Over 60 percent of respondents had clinically significant mental health symptoms, with the majority (59.7 percent) having scores suggestive of perceived stress. The greatest distress that patients reported was about their family’s health or contracting the COVID-19 virus themselves. Nearly three-quarters of respondents had increased stress overall, including stress regarding finances (37.5 percent) or the workplace (44.6 percent).

Alternatively, a survey of persons with IBD in the UK reported that the rates of depression, anxiety, and stress were common, although the frequency was similar to pre-pandemic rates.^36^

While mental health symptoms were high for everyone, those with chronic illnesses as well as those without, during the COVID-19 pandemic, it is unknown whether they were higher for persons with IBD compared with people who do not have IBD. Many of these symptoms were elevated initially and seemed to taper off as the pandemic continued, although it is unknown whether the reason for this is due to the introduction of vaccinations or acclimating to the pandemic. It is also unknown whether a subset of persons with IBD, particularly those who were immunosuppressed or were on corticosteroids, continued to have clinically significant mental health symptoms throughout the pandemic and whether these symptoms improved as the pandemic progressed. While many of the studies contain one-time surveys as opposed to longitudinal follow-up, these results reveal the increased stressors and anxieties that persons with IBD had during the pandemic, especially early on.

## Health care utilization through the pandemic

As hospitals started to admit patients affected by the COVID-19 infection, it was thought that this would lead to a decrease in hospitalizations and procedures for persons with IBD, as they attempted to avoid contracting the virus from nearby sick people.

In the USA, emergency room visits decreased in the early stages of the pandemic for the general population.^37^ Many surgical procedures were also cancelled or delayed in the early stages of the pandemic to free up resources.^38,39^ An observational study of two large centres in the USA showed emergency room visits dropping in the first year of the pandemic compared with the year prior to the pandemic, with more visits for patients with UC compared with CD.^40^ IBD-related hospitalizations decreased in one of the hospitals, but was unchanged in the other hospital, when compared the first year of the pandemic with the year prior. Overall IBD-related endoscopies and surgeries were unchanged between the 2 years. A database search in the Netherlands showed that both IBD-related surgeries and endoscopies decreased in the early months of the pandemic (February–August 2020).^41^ New diagnoses of IBD (based on their pathology database) also decreased by 6.5 percent compared with the previous two years, along with a decrease in indefinite and low-grade dysplasia diagnoses. A follow-up study of this retrospective cohort over 2 years revealed a net decrease in IBD procedures in the first 2 years of the pandemic compared with the 2 prior years, mostly comprised of endoscopic procedures.^42^ The highest reduction was early on in the pandemic in April 2020. There was a net increase in both endoscopic and surgical procedures in the second year of the pandemic compared with the first year. A decrease of 0.9 percent was seen in new IBD diagnoses in the first 2 years of the pandemic compared with the 2 prior years, with a net reduction of 1.9 percent in indefinite/low-grade dysplasia diagnoses in persons with IBD. There was no change in diagnoses of high-grade dysplasia or colorectal cancer.

Using administrative data in England, Deputy et al. showed that the first year of the pandemic saw a decrease in emergency room visits for both UC and CD compared with the 5 years prior, with higher rates of readmission to the emergency room within 28 days for acute UC.^43^

In summary, the COVID-19 pandemic saw a change in health care utilization for persons with IBD. Initially, a decrease in procedures in both endoscopic and surgical was seen. This rebounded in the second year as hospitals and health care centres adapted to pandemic rules and guidelines. A decrease in IBD diagnoses was seen in the Netherlands, perhaps due to delayed or cancelled endoscopic procedures, or persons avoiding the health care system altogether. Further research is needed to understand the long-term effects in persons with IBD of this initial decrease in health care utilization, including whether an increase in complications evolves such as an increase in surgical procedures or long-term colon cancer due to a decrease in endoscopic procedures and delayed management.

## Perceptions on access to care

As the COVID-19 pandemic began, in-person interactions were limited to try and curb transmission of the COVID-19 virus. This dramatically shifted how health care providers delivered health care, using virtual telemedicine (telephone or video), due to strict physical distancing and the use of personal protective equipment. Interestingly, Crohn’s and Colitis Canada had previously developed a telemedicine program in Ontario to deliver appropriate and timely care to persons with IBD, resulting in significant cost savings for those who live in Northern Ontario.^44^ During the early phase of the pandemic, the use of telemedicine was associated with increased satisfaction and efficiency.^45^

In our survey study of persons with IBD within Manitoba, 67.2 percent felt that they had good access to their primary care provider to discuss their non-IBD health care issues, while 56.5 percent felt that they had good access to their gastroenterologist to discuss their IBD, although this still left a large proportion who did not perceive ready access.^30^ Of these respondents 46.0 percent felt increased stress about accessing their doctor or nurse, while almost one-fifth had increased stress about accessing prescription therapy for their IBD. A study assessing participants from this registry in 2017 found that 42 percent reported having good access to their gastroenterologist by telephone when needed, hence 56 percent is actually an increase over pre-pandemic patient satisfaction rates.

Chen et al.^24^ revealed in their survey that over a half (58 percent) of the persons had increased worry about accessing their physician during the early stages of the pandemic. Another survey of health care providers and persons with IBD during the first year of the pandemic showed that the majority of health care providers (64 percent) surveyed did not believe their patients with IBD could keep up with routine follow-up, while only one-fifth of patients believed this to be true.^46^ Approximately half of the health care providers and patients surveyed felt that patients were very much or extremely involved in shared decision-making, with the major reason some patients were not involved was insufficient time, although they did not state if telemedicine care replacing in-person care was the reason for this. A half of the patients planned to use telehealth in the future. Another survey from Australia revealed that 88.1 percent of persons with IBD was satisfied or very satisfied with health care delivered through telemedicine, with 60 percent of respondents taking time off from work for an in-person appointment, compared with only 20 percent for a telemedicine appointment.^47^ Rates of non-attendance during the pandemic were similar to before the pandemic.

A prospective study of persons with IBD who underwent telephone consultation in the early stages of the pandemic revealed that 94.6 percent perceived it was effective, although nearly a half had preferred in-person follow-up.^48^ A cross-sectional survey on patient experiences with IBD revealed that most visits could be converted to telephone consultations, which lead to a high rate of patient satisfaction, and many of the patients preferred telephone visits over in-person visits, in the early phases of the pandemic.^49^

A literature review of telemedicine across many different medical specialities revealed that many persons enjoyed the lesser travelling time, and less waiting time, with better accessibility and found it more convenient. Drawbacks to telemedicine included technical difficulties and lack of a physical examination.^50^ Interestingly, Storan et al.^51^ conducted a survey of persons with IBD finding satisfaction with telemedicine varied based on the persons’ personality, as those with an open personality (intellectual curiosity and preference for variety), had a higher satisfaction with a virtual clinic, while persons with active disease and an agreeable personality (incorporating warmth, kindness, and cooperation) had lower satisfaction levels, perhaps indicating a need for personal interaction in these people.

Access to care during the COVID-19 pandemic quickly adapted virtual methods of communication, with many positive attributes. Persons with IBD appreciate the avoidance of often unnecessary travel to see their health care provider, as well as having to take time off of work. While many persons with IBD did have increased stress about accessing their primary care provider or gastroenterologist, the majority of these people had good access. Many persons with IBD still prefer an in-person component to their care, although many IBD clinics have evolved to incorporate both in-person and virtual components into their practice. It is also felt that virtual care can more easily be delivered to under-served and minority populations with IBD, helping to eliminate racial disparities in care.^52^ This also has cost savings, as persons who live far from in-person care, can instead receive care from their home, saving travel costs.

While many persons with IBD had increased stress about accessing their gastroenterologist during the pandemic, access to health care providers can improve with the development of telemedicine and lead to increased communication between patients and their IBD providers, as patients could access their gastroenterologist from home or work more frequently.

## Conclusion

The COVID-19 global pandemic significantly altered everyday life, from periods of isolation and quarantines to closures of public spaces, and changes in how health care was delivered. Persons with IBD are at an increased risk of mental health disorders, and during the pandemic an increase in clinically significant mental health symptoms was observed in these people as well as in the general population. While clinically significant mental health symptoms were also seen in the general population, it is unknown whether persons with IBD had worsening mental health symptoms over and above that was seen in the general public.

Persons with IBD also had increased stress about accessing their gastroenterologist and IBD medications during the pandemic, as many clinics shifted toward telemedicine to help deliver care. Much of this increased stress was early in the pandemic though, and persons with IBD were often satisfied with telemedicine as an option to communicate with their gastroenterologist, as it allowed them to communicate more frequently, and decrease travel time to clinics.

It is unknown whether the increase in mental health symptoms will continue as the pandemic fades, or lead to worsening outcomes with their IBD. It is also unknown whether the closures early in the pandemic will ultimately lead to an increase in surgical procedures in the future or an increase in neoplasia rates due to missed or delayed procedures. More research is needed to understand how clinically significant changes in health care access and delivery over a relatively short period of 1–2 years will affect long-term mental health, health care utilization, and long-term outcomes in persons with IBD.

## Data Availability

There are no new data associated with this article.
